# Rheoreaction impacts dispersal of fish larvae in restored rivers

**DOI:** 10.1002/rra.3630

**Published:** 2020-04-15

**Authors:** Martin Glas, Michael Tritthart, Hubert Keckeis, Aaron Lechner, Marcel Liedermann, Helmut Habersack

**Affiliations:** ^1^ Institute of Hydraulic Engineering and River Research, Department of Water, Atmosphere and Environment BOKU—University of Natural Resources and Life Sciences Vienna Vienna Austria; ^2^ Christian Doppler Laboratory for Sediment Research and Management, Institute of Hydraulic Engineering and River Research, Department of Water, Atmosphere and Environment BOKU—University of Natural Resources and Life Sciences Vienna Vienna Austria; ^3^ Department of Limnology and Bio‐Oceanography University of Vienna Vienna Austria; ^4^ Abteilung II ‐ Wirtschaft und Umweltschutz District Authority Bludenz Vorarlberg Austria

**Keywords:** movement pattern, active‐passive, behaviour, hydrodynamics, settlement, trajectories

## Abstract

Connectivity of nurseries and spawning habitats for young of the year life stage is essential for successful recruitment of fish populations and therefore provides a key indicator for river restoration measures. Models for dispersal offer the potential to draw conclusions regarding restoration scenarios and to fill knowledge gaps about possible implications for fish populations. A newly developed rheoreaction‐based correlated random walk model (RCRW), in combination with a three‐dimensional numerical model and a non‐steady‐state particle tracing model, was applied for nase carp larvae (*Chondrostoma nasus*) before and after a restoration project on the river Danube, Austria. Spatio‐temporal patterns of dispersal of virtual larvae, attached with rheoreactive behaviour, were analysed within both scenarios. In comparison to the heavily modified river reach, the restored reach enabled a greater amount of upstream movement from the release site and showed a generally higher variability of spatio‐temporal distribution patterns. In contrast, estimated total settlement of rheoreactive larvae was substantially higher for the situation prior to the restoration measure. By comparing model results with a previously field experiment it was found that model simulations including rheoreaction as a single behaviour for navigation could not explain the whole pattern of larval dispersal. Therefore it is highly recommended for future studies to develop larval dispersal models by considering other factors (i.e., behaviour, bio‐energetics and environmental factors) of existing and future individual‐based models, which could serve as a tool to analyse the effect of restoration measures for recruitment of riverine fish populations.

## 1 INTRODUCTION

River restoration measures, aiming to improve ecological conditions, have been widely implemented to overcome the extensive anthropogenic hydro‐morphological degradations, undertaken over recent centuries associated with land use change, flood protection, navigational aspects, and hydro‐electric damming. Young of the year stages of fish (YOY), which are heavily affected by these activities, are known to represent a bottle‐neck in terms of recruitment and therefore year‐class strength of fish populations (Schiemer, Keckeis, & Kamler, 2002). Besides the valuable implementation of field experiments, modelling represents a key tool to evaluate the effects of various scenarios of restoration measures on these vulnerable life stages. However, by assessing such restoration measures solely with the help of habitat models (e.g., Hauer, Unfer, Schmutz, & Habersack, 2008), the connectivity of potential spawning and nursery habitat is neglected. Individual‐based models (IBMs) for YOY fish, aiming to evaluate habitat connectivity, provide a useful tool to overcome this gap. These approaches are widely used in marine environments (Codling, Hill, Pitchford, & Simpson, 2004; Paris, Chérubin, & Cowen, 2007; Staaterman, Paris, & Helgers, 2012; Vikebø, Jørgensen, Kristiansen, & Fiksen, 2007), but still represent a new field of rivers science (Wolter & Sukhodolov, 2008).

Rheoreaction, defined as the fish's behaviour induced by the current (Pavlov, Kostin, Zvezdin, & Ponomareva, 2010), is known to be a driving factor for larval dispersal in rivers. Behavioural aspects of fish larvae were studied recently for riverine (Humphries & King, 2003; Lechner et al., 2014; Schludermann, Tritthart, Humphries, & Keckeis, 2012; Zens, Glas, Tritthart, Habersack, & Keckeis, 2018) and marine YOY species (Leis, 2006; Leis & Carson‐Ewart, 1997), showing that they considerably effect habitat connectivity. Lechner et al. (2014) encountered enhanced drift, settlement and habitat connectivity of YOY fish for a near‐natural shoreline, when compared to a heavily modified one. Schludermann et al. (2012) identified a significantly higher amount of recaptured settled larvae remaining upstream of a release site compared to the proximate areas downstream. Reichard, Jurajda, and Smith (2004) found larval drift maxima close to the shoreline but nevertheless drift was recorded in the main channel as well. Keckeis, Frankiewicz, and Schiemer (1996) and Loisl, Singer, and Keckeis (2014) determined the highest fish densities on gravel bars, representative for a typical natural hydro‐morphological condition, during the reproductive period. Lorenz, Stoll, Sundermann, and Haase (2013) detected significantly higher densities of YOY fish due to restoration measures. On the other hand Keckeis (2014) found contradictory short‐term effects of a restoration measure in the Danube River on the sublittoral assemblage as well as on the inshore areas (larvae). Although the abundance of larvae decreased the abundance of the sublittoral fish community increased immediately after implementing the restoration measure. In this context, White, Gerken, Paukert, and Makinster (2010) and Eick and Thiel (2013) suggest that river engineering structures may improve habitats for YOY fish, but a broader view considering the whole life cycle of fish is needed.

The nase carp (*Chondrostoma nasus*), a rheophilic cyprinid widespread in European rivers (Lelek, 1987), is often used as a target species in fish‐ecology studies (Keckeis et al., 1996; Ovidio & Philippart, 2008; Pichon, Tales, Gorges, Baudry, & Boët, 2016). The females spawn demersal eggs which developed within the gravel substrate. After hatching the larvae exhibit a benthic lifestyle before they swim up and disperse upstream or downstream aiming at finding suitable nursery habitats.

In contrast to marine areas, analyses of the connectivity between hatcheries and nurseries of YOY fish within inshore habitats in rivers by means of analytical or numerical tools are lacking so far. Due to the complexity of spatio‐temporal dispersal processes, such tools provide advantages to separate the factors and cues and to draw conclusions on various scenarios of restoration measures prior to their implementation in the field. In this study, a rheoreaction‐based larval model, developed in an experimental flume (Glas et al., 2017), was compared to dispersal patterns observed in the field (Lechner et al., 2014). Moreover, the influence of rheoreaction on dispersal of early life stages was investigated for two different scenarios, a heavily modified shore and a restructured, near natural gravel bar (before‐after comparison).

## 2 MATERIAL AND METHODS

### 2.1 Study site

**FIGURE 1:**
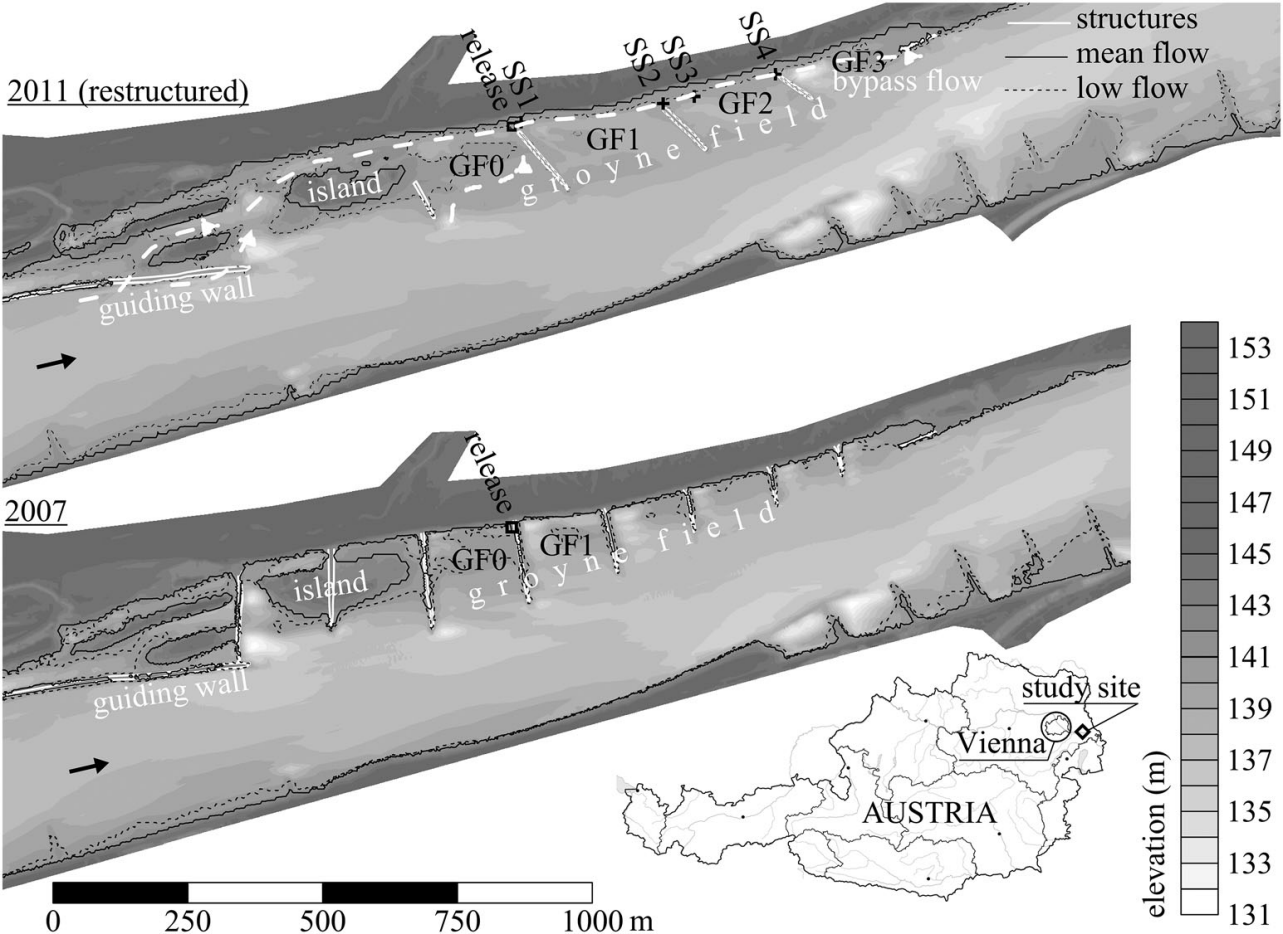
The hydro‐geomorphological situation of the investigated river reach before (2007) and after (2011) restoration. A digital elevation model (derived from Airborne Laserscan, Echolot bathymetry and tachymetric surveys) is presented in combination with water lines for approximately low flow (*Q* = 1,024 m^3^/s) and mean flow conditions (*Q* = 1,930 m^3^/s) as well as relevant river engineering structures. Groyne fields (GF0–GF4) are labelled with respect to the release sites. Furthermore, release sites and sampling sites (SS1–SS4) are given

The study was conducted in the national park “Nationalpark Donauauen, http://www.donauauen.at” at a river reach within the free flowing section of the Austrian Danube east of Vienna—near the village Witzelsdorf—between river‐km 1891.0 and 1894.0 (Figure 1). Mean discharge at the study site is *Q* = 1930 m^3^/s and low discharge (94% probability of exceedance) is *Q* = 980 m^3^/s. In the scope of an integrative restoration measure, the hydro‐geomorphological characteristics were adjusted at the left shore to improve ecological and navigational demands (Klasz, Schmalfuß, & Schlögl, 2008). The scenarios before (2007) and after (2011) the restoration measure, conducted from 2009 to 2010, were considered in this study (Figure 1). The groyne layout was adapted from a former orthogonal layout to an attracting layout. Groyne heights, ranging between a crest height equals to the water level at mean flow conditions, were reduced to a crest elevation equal to low flow water levels (+0.30 m). Groyne spacing of approximately one times the average groyne length was increased to a spacing twice the average groyne length. In order to establish a bypass flow along the shoreline at low flow, groyne roots were lowered to a water level below the low flow water levels. This adaption, in combination with a lowered crest elevation of an upstream guiding wall, increased the proportion of discharge in the groyne field and along the shoreline during mean flow by 6% (Habersack, 2013). The rip rap along the shoreline was removed and thus a near‐natural gravel bar was formed as a consequence of the bank erosion, the increased flow along the shoreline and the new attracting groyne layout.

### 2.2 Numerical models

#### 2.2.1 Three‐dimensional numerical model

The three‐dimensional (3D) numerical model RSim‐3D (Tritthart, 2005) iteratively solves the Reynolds‐averaged Navier–Stokes Equation with the help of a finite volume approach and a k‐ε model for turbulence closure. Details of the model approach are given in Tritthart and Gutknecht (2007). In our study the mesh distance of the 3D numerical model was set to 20 m in the main stream and refined sufficiently (down to 1 m) at structures, groyne fields and shorelines. Calibration and validation of the flow fields were applied successfully with the help of measured water levels, flow velocities and turbulent kinetic energy (Glas, Glock, Tritthart, Liedermann, & Habersack, 2018).

#### 2.2.2 Steady state and unsteady particle tracing model based on Tritthart et al.

Based on the calculated flow field of the 3D numerical model, trajectories of numerical particles were calculated with local temporal mean flow velocities and velocity fluctuations. According to the random walk approach employed, velocity fluctuations were derived from turbulent kinetic energy in combination with two random numbers between 0 and 1, each of them representative for one of the Cartesian coordinates *x* and *y*. Since fluctuations in the direction of the vertical coordinate *z* are usually one order of magnitude smaller than in the other Cartesian coordinates, the velocity in vertical direction was represented by its deterministic component only. The model was validated successfully in a groyne field with the help of spherical tracers. Details of the particle tracing model are presented in Tritthart, Liedermann, and Habersack (2009). In terms of the unsteady particle tracing model, flow fields were calculated for a number of steady‐state conditions sufficient to cover the desired hydrograph in a first step. Then, while running the particle tracing model, representative flow fields along the hydrograph were taken into account to calculate particle trajectories. Further information on the unsteady particle tracing approach is available in Tritthart, Gmeiner, Liedermann, and Habersack (2019).

#### 2.2.3 RCRW model based on Glas et al.

This 2D model approach was developed in the framework of a flume study, considering rheoreaction as a driving factor for dispersal of nase carp larvae. With the help of a raster based analysis, observations were categorized after Pavlov (1994) to derive the following movement patterns: “active downstream” (orientation equal to the flow and speed was greater than the flow velocity), “active‐passive” (orientation against the flow and speed was less than the flow velocity) and “passive” (random orientation and speed was equal to flow velocity). A fourth one (“active upstream”: orientation against the flow and speed is higher than flow), introduced by Zens et al. (2018), was considered as well. The RCRW model generates those movement patterns as well as durations within certain movement patterns (as derived from observations as well) over the simulation time. Horizontal swimming, based on the biased and correlated random walk model (Codling et al., 2004), was altered in terms of the inherited bias. To reproduce Pavlov's types of movement patterns, the preferred movement direction in the RCRW model was changed to be in accordance with the local flow direction. An additional user‐defined angle was introduced to shift the preferred movement direction towards the shoreline. Furthermore, horizontal swimming speed was derived from mean flow velocity and movement pattern. Further details of the model approach are given in Glas et al. (2017).

### 2.3 Experiments

In 2011 (restored scenario), nase carp larvae, marked with a fluorochrome dye (Alizarin Red S, ARS; Sigma Aldrich®) were released in the river close to the shoreline at dusk, according to the natural circadian peak in dispersal activity (Reichard, Jurajda, & Ondračkovaá, 2002; Zitek, Schmutz, & Ploner, 2004). Stationary nets, set to capture dispersing individuals, were placed at four consecutive sampling sites (SS1 to SS4) three to five times per evening with a sampling time of 0.5 hr. In addition, point abundance sampling (PAS) was undertaken equidistantly along the whole shoreline, in order to capture settling individuals. The sampling procedure started on the day of release and was repeated 2 and 5 days thereafter. Details of the methods, study design and results are given in Lechner et al. (2014, 2018). This mark‐recapture study on larval dispersal was accompanied by laboratory experiments, where movements of individual nase carp larvae were tracked continuously with a video camera to obtain trajectories, movement patterns, drift distances and, thus, data for the RCRW model approach. Details of the flume experiment are given in Glas et al. (2017) and Zens et al. (2018).

In this study, the field experiment was reconstructed numerically (with the help of depth averaged data calculated from the results of the fully 3D‐numerical model RSim‐3D and the RCRW model) in terms of the release site, the drift net sampling and the observed hydrograph (rise from low flow *Q* = 1,024 m^3^/s to approximately mean flow *Q* = 1,743 m^3^/s in 7 days). This observed hydrograph was extended by a theoretical increase (rise to approximately *Q* = 2,360 m^3^/s and fall to *Q* = 2,289 m^3^/s within 1 day) to reach almost full submergence of groynes in both scenarios (2007, 2011) 8 days after release. Nase carp larvae, associated with a developmental stage L4 after Peňáz (1974), were considered in the RCRW model. Thirteen steady‐state flow fields were calculated a priori with the 3D‐numerical model to approximate the considered hydrograph (discharge classification with natural breaks after Jenks & Caspall, 1971) for use in the unsteady particle tracing model. Parameterization of the RCRW model was obtained from the results of the validated flume study (Glas et al., 2017): turning rate *λ* = 2.0; orientating ability *κ* = 4; sensing ability *d*_*τ*_ = 0.6; altered preferred direction *θ*_*c*_ = 45°; scale factors for swimming speed *R*_Ap_ = 4.05 and *R*_*S*_ = 0.2; maximum swimming speed *S*_max_ = 0.175 m/s; minimum swimming speed *S*_min_ = 0.01 m/s. Input tables, according to the flow velocity dependent types and durations of movement patterns, were taken for larvae that are dedicated to an offshore release site within the flume, as this release site was more appropriate to the flow conditions at the release site in the field. The type of movement pattern was assumed to be “passive” at locations with flow velocities higher than 0.225 m/s.

For the numerical reproduction of the mark‐recapture study in 2011, 3,000 virtual larvae were released. Virtual drift nets were applied at the sampling sites (SS1 to SS4) according to the schedule of the field experiments. Recapture rates of virtual individuals (RR) per 1,000 m^3^ water were calculated for each net, as outlined in Lechner et al. (2014). In order to assess the restoration measure (2011), the numerical experiment was repeated, accordingly, for the situation prior to implementation of restoration measures (2007, number of released virtual larvae: *n* = 1,700), omitting the application of virtual drift nets. Furthermore, passive particles (*n* = 100) were released in both scenarios (2007, 2011) in order to provide a reference. Spatial distributions of larvae at specific times (3, 5 and 7 days after release) as well as times of larvae and particles leaving the river reach at the downstream end were evaluated for both scenarios (2007, 2011). In addition, trajectories of virtual larvae were categorized in terms of four typical paths (A, B, C, D; Table 1) with the help of the calculation of intersections of trajectories with analysis polygons along relevant groynes, lowered groyne roots and river sections in the main stream. Finally, differences in proportions of typical paths for both scenarios (2007, 2011) were analysed.

**TABLE 1:** Description of the investigated path types

Typical path	Description
A	Larvae leaving the groyne field GF1 (2011) towards the main channel
B	Larvae passing the first and the second lowered groyne root downstream the release and leaving the groyne field (GF2) either towards the main stream or passing the groyne to GF3
C	Larvae passing all lowered groyne roots downstream the release and leaving GF3 towards the main stream
D	Larvae performing upstream movement with respect to the release site, settlement on the shoreline of the island or the left shore
*D*_B_, *D*_C_	Larvae performing upstream movement with respect to the release site, followed by passive drift over the groynes or through the lowered groyne roots, until reaching the exit of the area downstream according to path B (*D*_B_) or C (*D*_C_).

## 3 RESULTS AND DISCUSSION

### 3.1 Shoreline configurations

#### 3.1.1 Hydrodynamics

**FIGURE 2:**
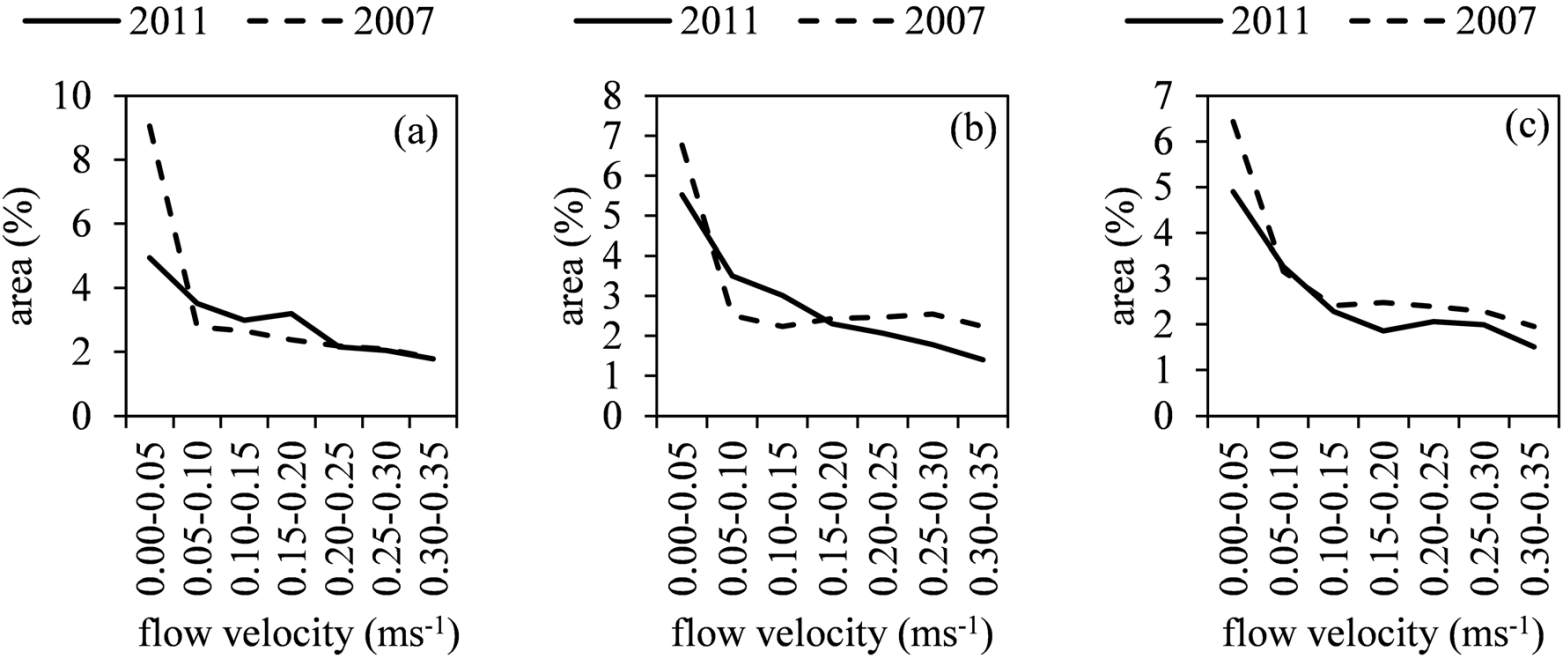
Comparison of the areal flow velocity distribution (%) between the scenarios 2007 and 2011 for (a) *Q* = 1,024 m^3^/s, (b) *Q* = 1,743 m^3^/s and (c) *Q* = 2064 m^3^/s

The hydrodynamics for emerged groynes in 2011 (*Q* < 1,150 m^3^/s) was characterized by two counter‐rotating cells in the groyne fields and a parallel flow along the shoreline due to the introduced bypass flow. Submerged groynes induce flow perpendicular to the attracting groynes. In 2007, single counter‐rotating cells occurred when groynes were emerged (*Q* < 1930 m^3^/s) and flow perpendicular to the orthogonal groyne structures in combination with small and single counter‐rotating cells close to the shore was developed for partially submerged groynes (1930 m^3^/s^1^ ≤ *Q* ≤ 2,500 m^3^/s). Hence, flow velocity characteristics, shown in Figure 2, differed between investigated shoreline configurations. For all analysed discharges, the restored scenario of 2011 showed fewer areas with low flow velocities up to 0.05 m/s than the scenario of 2007, due to increased flow along the shoreline. In contrast, areas of flow velocities between 0.05 and 0.20 m/s increased following restoration measures.

#### 3.1.2 Restored geomorphological situation (2011)

**FIGURE 3:**
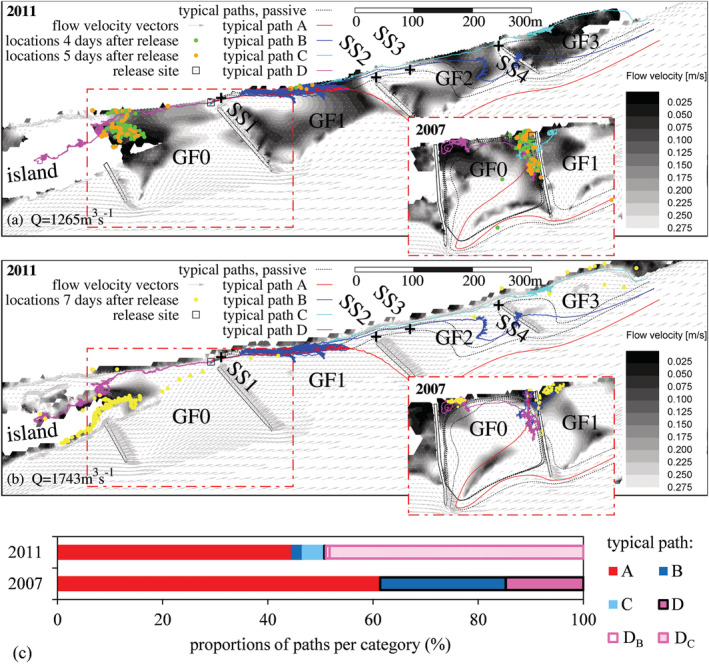
Spatial distribution of virtual rheoreactive larvae and passive particles: (a) spatial distribution of larvae 3 and 5 days after release and typical paths for scenarios 2007 and 2011 (*Q* = 1,265 m^3^/s); (b) spatial distribution of larvae 7 days after release and typical paths for scenarios 2007 and 2011 (*Q* = 1,743 m^3^/s) and (c) proportions of typical paths for scenarios 2007 and 2011. Proportions of settled larvae 8 days after release are outlined with black lines [Colour figure can be viewed at wileyonlinelibrary.com]

Trajectories of the RCRW model (Figure 3(a) and (b)) were assigned to one of four typical path types (Table 1). Evaluated proportions of those path types are shown in Figure 3(c). In 2011, 49.3% of the released larvae exhibited an upstream movement with respect to the release site and the current direction (type D, D_B_ and D_C_). Larval upstream movements in the Danube River were also observed by Schludermann et al. (2012) and Lechner et al. (2014). Downstream dispersal with respect to the release site and current direction is accounted for 50.7% (types A, B, C), whereas most larvae left GF1 towards the main stream (44.6%, type A). Larval drift was recorded in the main stream by Reichard et al. (2004), too.

**FIGURE 4:**
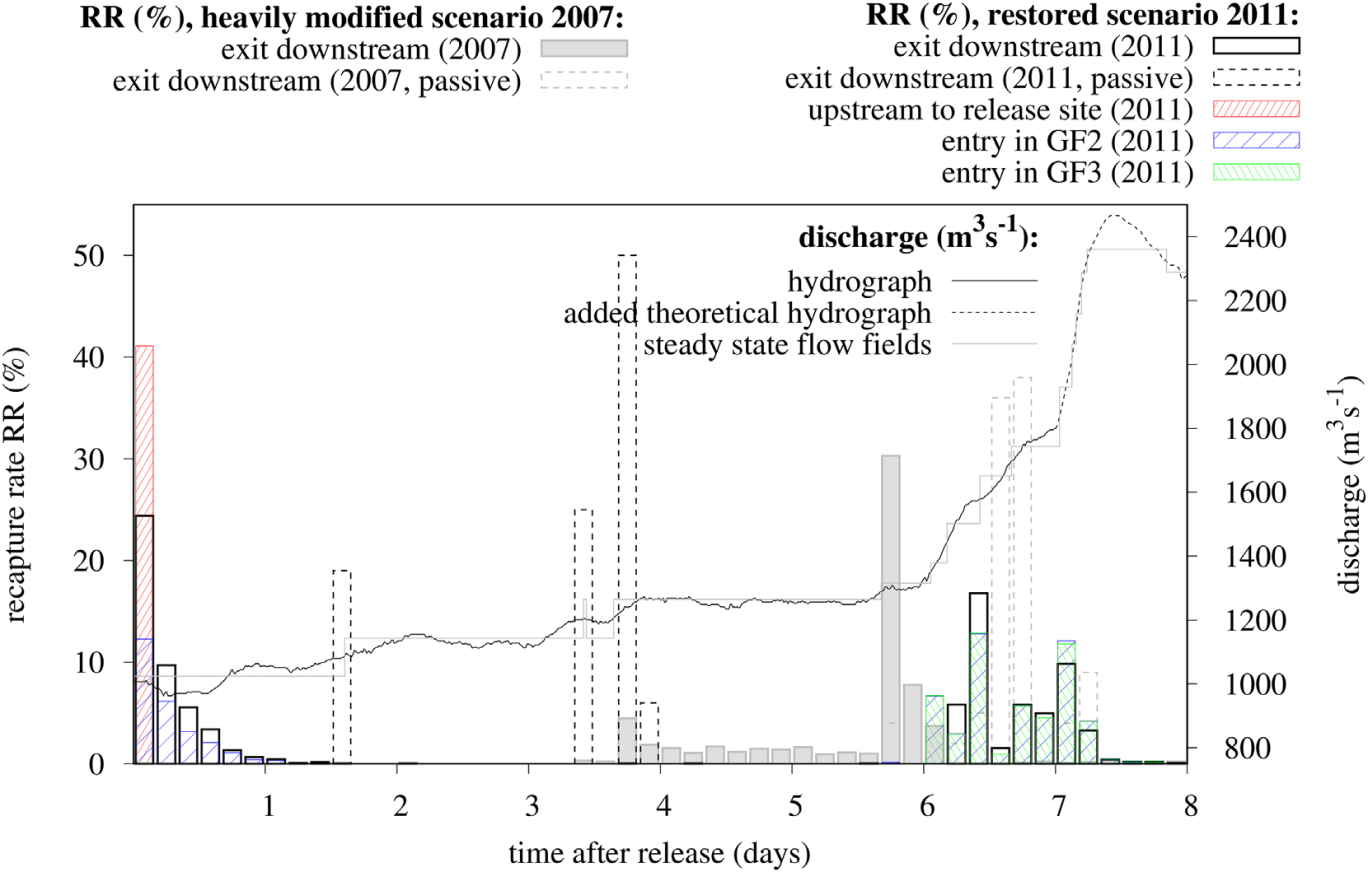
Temporal distribution of the RR (%) at certain locations (exit downstream, upstream to release site, entry in GF2 or GF3) indicated by boxes (box width: 4 hr) for scenarios 2007 and 2011. The considered hydrograph and the calculated steady‐state flow fields are referring to the second ordinate [Colour figure can be viewed at wileyonlinelibrary.com]

With respect to temporal variability, it was found that dispersing virtual larvae reached the exit of the observed area 1.5 days after release (Figure 4). Therefore, larvae can be related to types A, B and C, as outlined in the distribution of RR (%) in GF2 and GF3 within the same period (Figure 4). Repeated circular movements of virtual larvae at the shoreline in GF1—increasing retention temporarily—were observed for types B and C, comprising upstream movements along the shore and downstream dispersal in areas with higher flow velocities further away from the shoreline. This was in accordance with the flume experiment (Glas et al., 2017). Furthermore, higher fish densities on gravel bars (Keckeis et al., 1996; Loisl et al., 2014) were likely due to circular movements. Passive particles left the observed area substantially later than virtual larvae (path type B or C), as local flow velocities along the passive trajectories were notably smaller. Peaks of exit events occurred 1.6 and 3.7 days after release (Figure 4). It was obvious that the peaks were related to the instantaneous increase of the hydrograph.

Upstream movements of virtual larvae with respect to the release (types D, D_B_ and D_C_) occurred within the first 4 hr after release (Figure 4). Locations of those temporarily settling larvae dominated close to the island and along the shoreline 4 and 5 days after release (Figure 3(a)). Seven days after release (Figure 3(b)), according to the increased hydrograph, most of the those previously settling larvae either moved along the shoreline of the island facing the main channel or dispersed downstream, as indicated in exit events 6–8 days after release (Figure 4). 48.3% of those larvae took the bypass flow along the lowered groyne roots (type D_C_) and entered the main channel at GF3. The approximately equal distribution of RR for entries in GF2 and GF3 (Figure 4) suggests that most of the larva were drifting passively. However, 0.3% of larvae were located at the shorelines along GF3 (Figure 3(b)) 7 days after release. Finally, 8 days after release, 99.6% of released virtual larvae were washed out of the study area. Sparse settlement of larvae could be detected along the shoreline of the island facing the main channel.

As a consequence of increased lateral dispersal of virtual rheoreactive larvae, the chance for them reaching areas with higher flow velocities and thus being dispersed to the exit downstream, were increased. The peak in passive wash‐out of virtual larvae during increased discharge (6–8 days after release) was not reflected by the findings of Lechner et al. (2018) in the field, where drift rates seemed to be insensitive to discharge on the gravel bar. This contradiction might be due to the assumption of “passive” transport in the RCRW model, for flow situations when flow velocity exceeded *U* ≥ 0.225 m/s (as no data from the flume experiments was available for this case).

#### 3.1.3 Situation prior to restoration (2007)

In 2007, virtual larvae showed low dispersal distances (11.6 ± 0.2 m) during the first day after release (low flow, Q = 1,024 m^3^/s). Dispersal activity was restricted to a small pool, shaped by the higher crest elevations of groynes, which was eventually connected to the river at rising discharges approximately 1.5 days after release. In proximity to the release site, flow velocity, representative for the main trigger of larval swimming speed in the RCRW model, was low, ranging from 0 to 0.01 m/s. Thus, in 2007 exit events occurred at two maxima, 3.8 and 5.8 days after release (Figure 4), later than in 2011. Both peaks initiated periods of increased exit events, each lasting around 2 days. The added theoretical hydrograph 7 days after release did not substantially reduce larval settlement. Total settlement 8 days after release amounted to 37% in 2007. Therefore, larvae were settling along the shorelines and the groynes of both GF0 and GF1. As found by Lechner et al. (2014) and in contrast to the restored scenario (2011), the main proportion of larvae, which drift or disperse from the shore in the direction of the main channel (type A, Figure 3(c)), were not able to re‐enter groyne fields further downstream, especially because groynes were not fully submerged close to the shore for the hydrograph considered. The smaller proportion reaching GF1 (type B, Figure 3(b)), did not provide a source for dispersal towards the subsequent groyne field further downstream. Passive particles were characterized by later occurrences of exit events of the investigated area (5.8–8 days after release) than virtual larvae due to the low flow velocities in the area sheltered by the groynes. However, differences in the temporal occurrence of exit events were found to be smaller and less variable in 2007 than in 2011. Therefore, rheoreaction as a driving factor is less important in terms of larval dispersal for the situation of the heavily modified shoreline (2007).

### 3.2 Rheoreactive virtual larvae versus observed larvae (2011)

In terms of the reconstruction of the mark‐recapture experiment, the modelled drift rate of rheoreactive larvae was overestimated at nets applied during the first day (Table 2) when compared to the observed data. The opposite holds true for the following days, as no larvae were found in any of the virtual nets. Furthermore, modelled drift was observed at SS1 and SS3 (1 day after release) only, whereas observed drift occurred at all SS. Moreover, recaptured larvae from PAS (Lechner et al., 2014), representing larval settlement at inshore habitats, showed larvae at inshore areas along the whole stretch of the gravel bar during the whole experiment. In comparison, modelled rheoreactive larvae could only be found in the upstream region during this period. The difference between observed and computed larval drift rates and settlement rates suggests that either (a) the RCRW model neglects important factors operating in the field or (b) rheoreaction is not the only behaviour determining larval dispersal.

**TABLE 2:** Averaged number of larval drift (L4, inshore release) per SS and day after release (RR Ind./ 1000m^3^ filtered water) for observed (Lechner et al., 2014) and modelled (rheoreactive) cases

Day after release	Sampling site	Observed RR (Ind. / 1,000 m^3^ filtered water)	Modelled RR (Ind. / 1,000 m^3^ filtered water)
1	Total	0.361 ± 0.73	1.89 ± 7.32
	SS1	1.14 **±** 1.07	6.90 **±** 13.3
	SS2	0.0675 ± 0.141	0
	SS3	0.0679 ± 0.151	0.0389 ± 0.117
	SS4	0.0690 ± 0.169	0
2	Total	0.0464 ± 0.127	0
	SS1	0.0976 ± 0.180	0
	SS2	0.0232 ± 0.0389	0
	SS3	0.0617 ± 0.164	0
5	Total	0.0148 ± 0.0544	0
	SS3	0.0165 ± 0.0571	0
	SS4	0.0430 ± 0.08.93	0

Abbreviations: RR, Recapture rates.

## 4 CONCLUSIONS

Rheoreaction as a key behaviour for larval dispersal (Pavlov et al., 2010) was investigated for an integrative restoration project on the river Danube by means of a recently developed and validated RCRW model. Analytical methods for assessing the complex spatio‐temporal processes of numerical larval drift and dispersal were developed and presented. In comparison to the heavily modified river reach, the restored, near natural reach enabled a higher amount of upstream dispersal and a generally higher variability in terms of the complex spatio‐temporal distributions of larvae. In contrast, total settlement of rheoreactive larvae was substantially higher at the situation prior to the restoration measure. A possible explanation can be found in the theory of river engineering structures improving habitat conditions for YOY fish (White et al., 2010) for specific hydrographs. However, this may also raise questions in terms of habitat connectivity on heavily modified rivers. Also Keckeis (2014) found a contradictory short‐term effect of a restoration measure for nase carp larvae in the river Danube. However, a comparison of numerical larval drift and settlement with field‐based observations (Lechner et al., 2014) suggests that rheoreaction is important but not the only behaviour determining dispersal of larvae. Obviously, virtual larvae attached with rheoreaction behaviour were able to withstand the flow and increase inshore retention, but larvae were dispersed rapidly, when flow conditions locally increased. In comparison to passive particles, virtual rheoreactive larvae frequently changed the lateral position in relation to the shoreline and therefore were more often exposed to higher local flow velocities. Therefore, aiming for the development of an IBM for riverine fish larvae for future investigations, the following is suggested: (a) inclusion of a vertical swimming behaviour (Vikebø et al., 2007) and application of the model in a 3D flow field; (b) extension of the evaluation of movement patterns for flow velocities *U* ≥ 0.225 m/s and validation of the RCRW model in further flume experiments; (c) inclusion of a circadian drift maximum at dusk and, on the other hand, a promotion of settlement during the day; (d) implementation of variable instead of constant model parameters with regard to specific biotic or abiotic aspects; (e) inclusion of other settlement cues, as suggested by Lechner, Keckeis, and Humphries (2016), for example, acoustic or visual signals (Codling et al., 2004; Staaterman et al., 2012) or hydraulic gradients, indicating shore proximity; (f) determination and inclusion of the mode of drift entrance (Lechner et al., 2016), characterized by “active,” “coincidental” and “passive” modes; (g) investigation and inclusion of a potential effect of larvae hiding in proximity to the gravel (Holzapfel & Hauer, 2013) or the boundary layer (Nikora, 2010); (h) inclusion of further aspects affecting dispersal (e.g., growth, feeding, mortality, predation, vessel‐induced waves).

As a consequence, RCRW and IBM have the potential to serve as an important tool to assess river restoration measures in terms of habitat connectivity of YOY fishes, aiming to improve plannings of future restoration measures and to increase knowledge relating to some hydro‐morphological conditions.

## Data Availability

Data sharing is not applicable to this article as no new data were created or analyzed in this study.
